# Moisture Absorption/Desorption Effects on Flexural Property of Glass-Fiber-Reinforced Polyester Laminates: Three-Point Bending Test and Coupled Hygro-Mechanical Finite Element Analysis

**DOI:** 10.3390/polym8080290

**Published:** 2016-08-10

**Authors:** Xu Jiang, Jie Song, Xuhong Qiang, Henk Kolstein, Frans Bijlaard

**Affiliations:** 1 Department of Bridge Engineering, College of Civil Engineering, Tongji University, Shanghai 200092, China; jiangxu@tongji.edu.cn; 2 Shandong Provincial Academy of Building Research, Jinan 250031, China; shipgoing@126.com; 3 Department of Structural Engineering, College of Civil Engineering, Tongji University, Shanghai 200092, China; 4 Faculty of Civil Engineering and Geosciences, Delft University of Technology, Delft 2628CN, The Netherlands; M.H.Kolstein@tudelft.nl (H.K.); F.S.K.Bijlaard@tudelft.nl (F.B.)

**Keywords:** glass fibre reinforced polymer, bridge deck, environmental degradation, mechanical property, coupled hygro-mechanical numerical analysis

## Abstract

Influence of moisture absorption/desorption on the flexural properties of Glass-fibre-reinforced polymer (GFRP) laminates was experimentally investigated under hot/wet aging environments. To characterize mechanical degradation, three-point bending tests were performed following the ASTM test standard (ASTM D790-10A). The flexural properties of dry (0% *M*_t_/*M*_∞_), moisture unsaturated (30% *M*_t_/*M*_∞_ and 50% *M*_t_/*M*_∞_) and moisture saturated (100% *M*_t_/*M*_∞_) specimens at both 20 and 40 °C test temperatures were compared. One cycle of moisture absorption-desorption process was considered in this study to investigate the mechanical degradation scale and the permanent damage of GFRP laminates induced by moisture diffusion. Experimental results confirm that the combination of moisture and temperature effects sincerely deteriorates the flexural properties of GFRP laminates, on both strength and stiffness. Furthermore, the reducing percentage of flexural strength is found much larger than that of E-modulus. Unrecoverable losses of E-modulus (15.0%) and flexural strength (16.4%) for the GFRP laminates experiencing one cycle of moisture absorption/desorption process are evident at the test temperature of 40 °C, but not for the case of 20 °C test temperature. Moreover, a coupled hygro-mechanical Finite Element (FE) model was developed to characterize the mechanical behaviors of GFRP laminates at different moisture absorption/desorption stages, and the modeling method was subsequently validated with flexural test results.

## 1. Introduction

During the last two decades, fibre-reinforced polymer (FRP) bridge decks are increasingly being used for the rehabilitation of old concrete-steel composite bridges and new construction of pedestrian and highway bridges [1,2,3,4,5,6,7], due to their various advantages [8]: high strength to weight ratio, corrosion resistance, controllable quality, rapid installation with minimum traffic disruption and low maintenance cost. Although FRP decks are increasingly being employed in civil infrastructure applications, their durability and long-term performance are still not comprehensively understood. In the normal service life of such infrastructures, FRP decks are usually exposed to harsh and changing environments, involving large variations in temperature and humidity. The “hot/wet” exposure is supposed to be the severest environmental condition to degrade the performance of polymeric materials [9,10,11,12,13,14,15,16,17], which decreases the service-life of FRP composite bridges. The influence of moisture absorption on mechanical properties of FRP composites is well documented in literatures [9,10,11,12,13,14,16,17,18,19,20,21,22,23,24,25,26], regarding the tensile, interlaminar shear and flexural properties. Shao et al.’s research [9] found that the tensile strength of the aged composites decreased with the increase of percent absorption. In their test results, the reduction was estimated at about 60% for both the web and the flange pultruded sheet pile sections. In Nogueira et al.’s research [11], the effects of absorbed water on the mechanical properties of an amine-cured epoxy resin system were studied. The overall reductions of elastic modulus, elongation at break, tensile strength, tensile toughness, Rockwell hardness and glass transition temperature were recorded in their test results, which were attributed to the plasticizing effect of water. From the SEM image investigation of E-glass fiber reinforced vinyl ester composite after hygrothermal aging, the fiber-matrix interfacial bonding and consequently the stability of the composite were found highly sensitive to moisture absorption [24]. In Dell’Anno et al.’s research [17], the interlaminar shear and flexural performance of the carbon fibre composites with isophthalic polyester, vinyl ester and urethane acrylate matrices were investigated under the aging condition of 40 °C-deionised water and compared to those of equivalent composites impregnated with three grades of epoxy resin. Their results demonstrated that, although the epoxy systems perform equally or better than the alternative resins in the dry state, they are also more sensitive to property degradation due to water ingress.

However, most of available researches were conducted in the aerospace engineering field and automotive industry. For FRP composites used in the civil engineering field, the available research results are very limited. Although test data collected from other research fields can be suggestive, but it is not convincing to directly extend the experimental data and results to the civil engineering field. Since FRP composites used for civil infrastructures have essential differences, which include geometries, types of fibres and matrix, fabrication methods, curing process and service environmental conditions as well. In order to keep pace with the application of FRP composites in Civil Engineering field, this research was undertaken to reveal more knowledge about the environmental degradation of Glass-fiber-reinforced Polymer (GFRP) composite bridge under hot/wet environment. In GFRP bridge decks, the GFRP laminates are mainly loaded by the wheel load in the through-thickness direction. Therefore, the flexural properties of GFRP materials need to be carefully studied. Thus, in this paper, the influence of moisture and temperature on the flexural properties of GFRP laminates was experimentally investigated. Three-point bending tests were performed following the ASTM test standard (ASTM D790-10A). The flexural properties of dry (0% *M*_t_/*M*_∞_), moisture unsaturated (30% *M*_t_/*M*_∞_ and 50% *M*_t_/*M*_∞_) and moisture saturated (100% *M*_t_/*M*_∞_) specimens at the test temperatures of both 20 and 40 °C were compared. One cycle of moisture absorption-desorption process was also included in this study to investigate how the residual damage induced by the moisture diffusion degrades the mechanical properties of GFRP laminates. Furthermore, to better understand the environment-dependent mechanical performance of GFRP laminates, a coupled hygro-mechanical FE model was developed by writing a specific post-progressing subroutine to work together with the FEM software ABAQUS, and subsequently validated with the flexural test results.

## 2. Experiment

**Figure 1:**
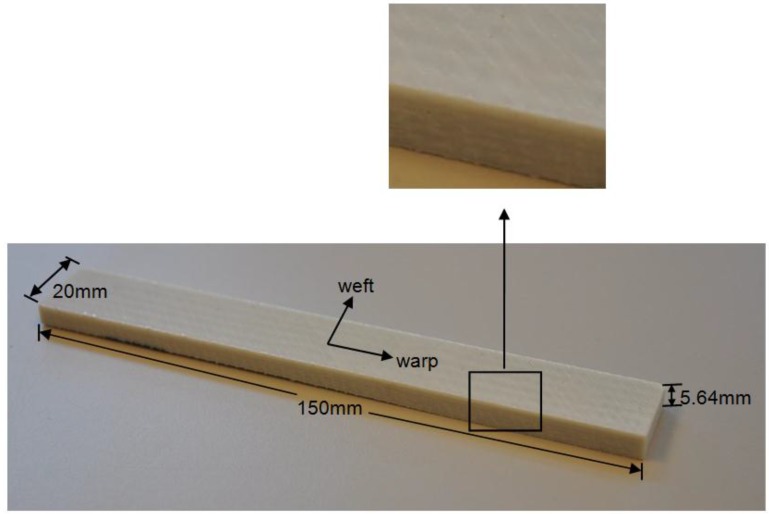
Fibre-reinforced polymer (FRP) laminate specimen for flexural tests.

To investigate the flexural property of GFRP laminates, three-point bending tests were employed. The whole test procedure follows the test code ASTM D790-10 [27]. GFRP laminates studied in this paper were manufactured by resin vacuum infusion (Infra Composite BV, Delft, the Netherlands) using polyester, and then cut into specific dimensions with the principal axis parallel to the warp direction of the woven roving (as shown in Figure 1). The glass transition temperature (*T*_g_) of GFRP laminates is 78 °C. The 5.64 mm thick specimen is composed of six layers of standard 0.94 mm EQX1200. The layup configuration of each piece of the standard 0.94 mm EQX1200 is illustrated in Table 1. The mechanical properties of GFRP laminates supplied by the manufacturer are shown in Table 2. The nominal length and width of the specimens were selected to be 150 and 20 mm respectively. According to the test code ASTM D790-10 [27], the specimen length shall be sufficient to allow for overhanging on each end of at least 10% of the support span. The specimen width shall not exceed one fourth of the support span for specimens greater than 3.2 mm in depth.

**Figure 2:**
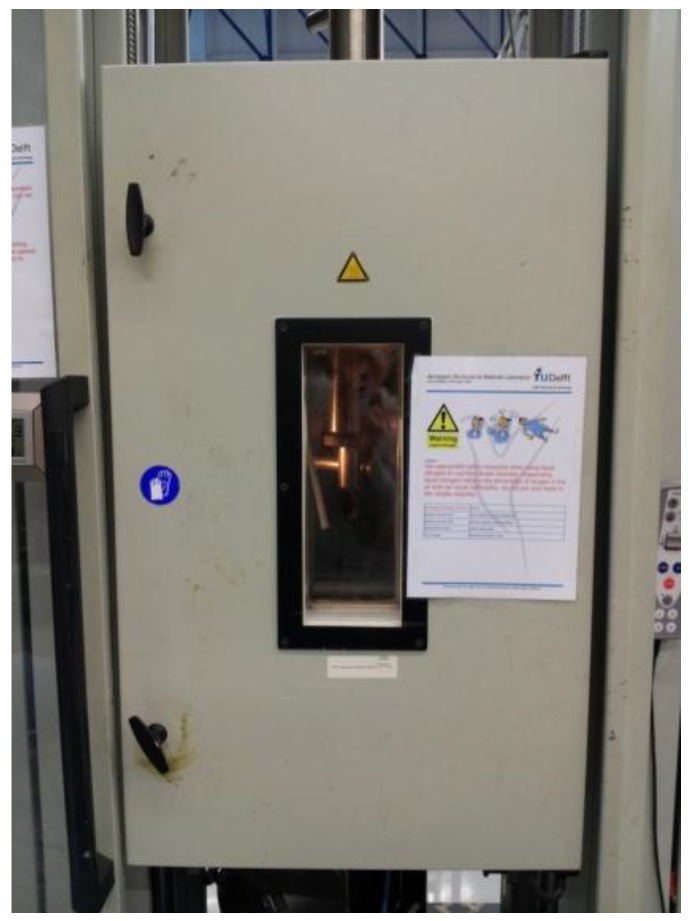
Climate chamber.

The numbering of specimens is given in Table 3, with regard to the moisture uptake content, test temperature, absorption/desorption process and replicated number. Two test temperatures 20 and 40 °C were selected for the three point bending tests, which were controlled by the climate chamber with the tolerance of ± 2 °C, as shown in Figure 2.

As listed in Table 3, the test in each condition was repeated five times to investigate the spread of test results. The hydrothermal aging condition was 40 °C-water, which is supposed to be a severe hot/wet condition for GFRP laminates, as discussed in previous research [28]. In total, 70 pieces of specimens were prepared. During the hydrothermal aging process, all the specimens were immersed in the water at 40 °C, except the F-0-20 °C and F-0-40 °C specimens, which are the as-received reference specimens (Set-1 in Table 3). The as-received specimens were stored in the laboratory environment. The moisture content of them is very low, and thus can be ignored. As illustrated in Table 3, the Set-2 specimens (F-30%-20 °C and F-30%-40 °C) were tested at 30% relative moisture uptake content. The Set-3 specimens (F-50%-20 °C and F-50%-40 °C) were tested at 50% relative moisture uptake content. The Set-4 specimens (F-100%-20 °C and F-100%-40 °C) were tested at the moisture saturation level (100% relative moisture uptake content). Until this time point, the above test process was considered as the moisture absorption process. Then, the remaining specimens were all taken out of the hydrothermal aging environment, and put into an oven at 42 °C to dry them, which was considered as the moisture desorption process. In this way, the Set-5 specimens (F-50%-20 °C-D and F-50%-40 °C-D) were tested at 50% relative moisture uptake content after a certain time of moisture desorption. Subsequently, the Set-6 specimens (F-30%-20 °C-D and F-30%-40 °C-D) were tested at 30% relative moisture uptake content after the moisture desorption. The final Set-7 specimens (F-0-20 °C-D and F-0-40 °C-D) were the fully dry specimens after one cycle of moisture absorption-desorption process. Herein, the symbol “D” indicates the moisture desorption.

**Figure 3:**
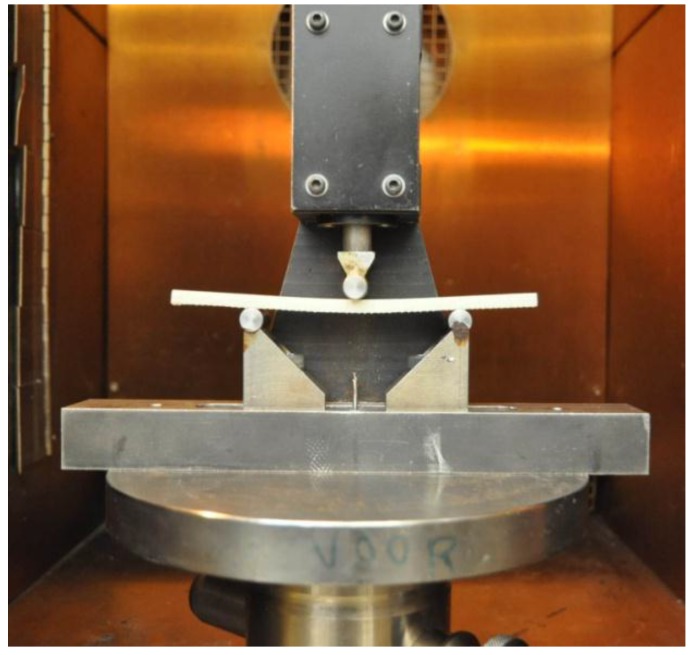
Flexural test device.

As shown in Figure 3, the whole three-point bending test set-up was put into the chamber. According to ASTM D790-10 [27], the support span-to-depth ratio is 16:1. Thus, the support span was proposed to be 90.24 mm, but it varied between different groups of specimens, since the value of the support span was exactly calculated based on the average real thickness of each group of specimens. The radii of the loading nose and supports were 5.0 ± 0.1 mm. The specimens were loaded at the strain rate 0.01 mm/mm/min. Correspondingly, the rate of crosshead motion was 2.4 mm/min, which is calculated as follows [27]:

(1) $$R=ZL^{2}\;/\;6d$$

*R* = rate of crosshead motion, mm/min, *L* = support span, mm, *d* = depth of FRP beam tested, mm, *Z* = rate of straining of the outer fiber, mm/mm/min.

**Figure 4:**
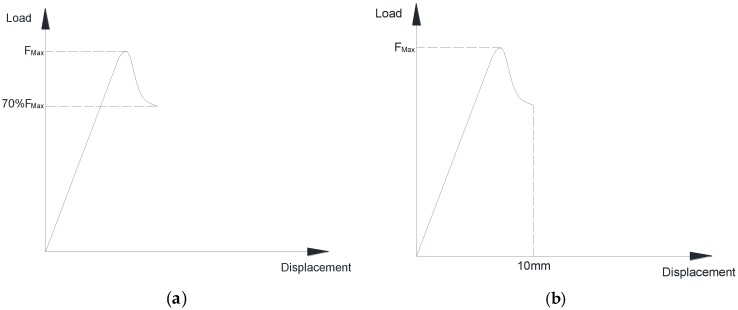
Termination rule of the flexural test. (**a**) Drop to 30% of the maximum load; (**b**) Maximum displacement of 10 mm.

The specimen was deflected until the load dropped to 30% of the maximum load or the maximum displacement of mid-span reached 10 mm, whichever occurred first (as illustrated in Figure 4). The experimental data was recorded per second. To track the moisture absorption process in GFRP laminate specimens, gravimetric tests were also conducted, the detailed test procedure of which can be found in previous research [28].

## 3. Results and Discussion

**Figure 5:**
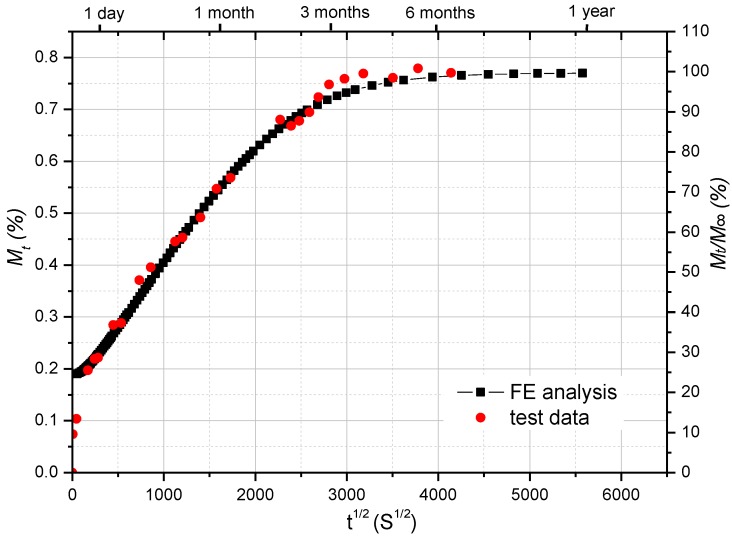
Comparison of moisture uptake curve between test results and FE analysis on FRP specimens for flexural test.

Figure 5 shows the moisture absorption process of GFRP laminate flexural test specimens immersed in water of 40 °C, compared with the FE moisture diffusion analysis. The moisture uptake content (*M*_t_) absorbed by each specimen was calculated according to its weight before exposure (*w_0_*) and after exposure (*w_t_*) as follows:

(2) $$M_{t}=100\times \left(\frac{w_{t}-w_{0}}{w_{0}}\right)$$

Moisture content is established as the function of the square root of time. It can be found that the moisture saturation level (*M*_∞_) is about 0.77%, which is in line with the gravimetric experimental results obtained in the previous research [28]. The specimens developed a similar moisture diffusion curve as that simulated by the FE analysis [28], which verifies the accuracy of the FE moisture diffusion model.

**Figure 6:**
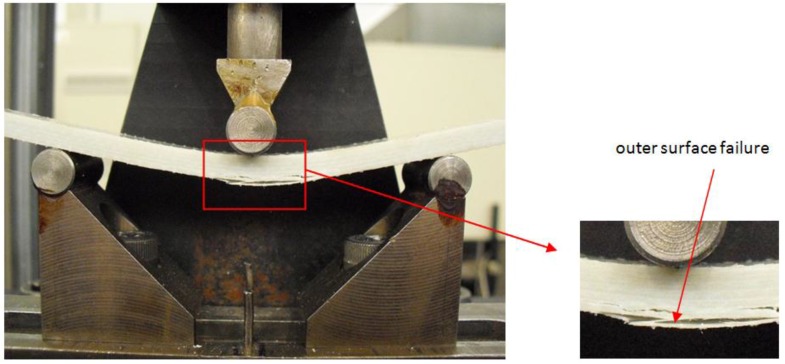
Failure mode of the flexural test specimen.

The typical failure mode of specimens under flexural tests is shown in Figure 6, where rupture occurs in the outer surface of the test specimen. In order to obtain the E-modulus of GFRP laminates, the stress and strain at the mid-span of GFRP specimens were calculated as follows.

According to ASTM D790-10 [27], the flexural stress in the outer surface of the specimen at midpoint was calculated by means of the following equation:

(3) $$\sigma =3PL\;/\;2bd^{2}$$

where: σ = stress in the outer fibers at midpoint, MPa, P = load at the mid-span on the load-deflection curve, N, *b* = width of FRP beam tested, mm.

The flexural strain, that nominal fractional change in the length of an element of the outer surface of the test specimen at mid-span, was calculated for any deflection using Equation (4):

(4) $$\varepsilon =6D_{\max}d\;/\;L^{2}$$

where: ε = strain in the outer fibers at midpoint, MPa, *D*_max_ = maximum deflection of the center of the beam, mm.

**Figure 7:**
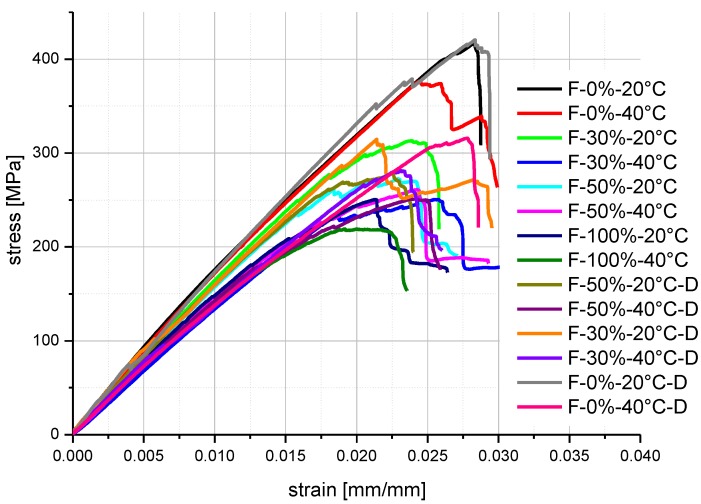
Stress-strain curves of FRP specimens under flexural tests.

The stress-strain curves are presented in Figure 7. To make the comparison more clear, the curve of only one specimen under each test condition is presented, which was selected visually as the average curve of the five specimens.

The E-modulus is represented by the chord modulus, which was calculated from two discrete points on the load-deflection curve, using Equation (5) [27]:

(5) $$E=(\sigma_{2}-\sigma_{1})\;/\;(\varepsilon_{2}-\varepsilon_{1})$$

$\sigma_{1}$, $\varepsilon_{1}$ and $\sigma_{2}$, $\varepsilon_{2}$ are the flexural stress and strain selected at two points of stress-strain curves (see Figure 7) in the linear stable range.

**Figure 8:**
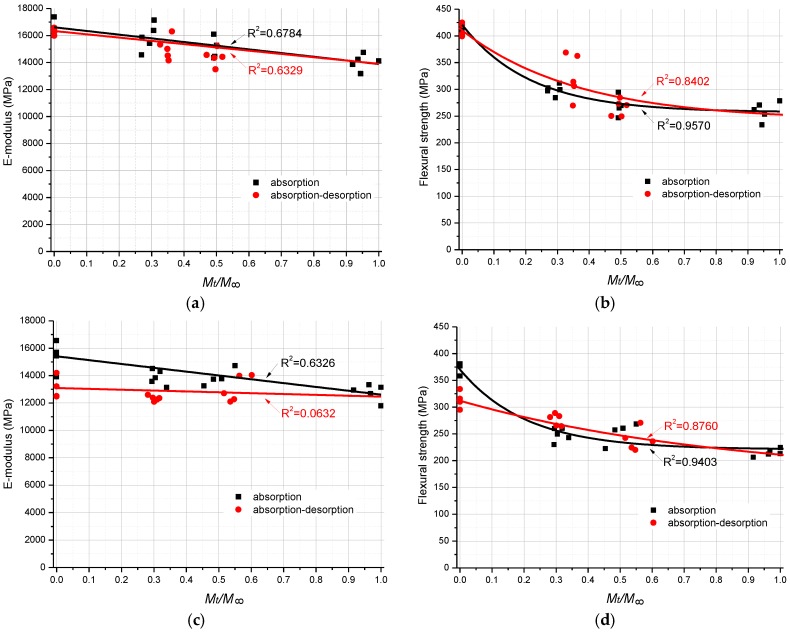
Environment-dependent flexural property degradation of FRP laminates. (**a**) E-modulus, 20 °C; (**b**) Strength, 20 °C; (**c**) E-modulus, 40 °C; (**d**) Strength, 40 °C.

The flexural strength is the maximum flexural stress sustained by the test specimen during the flexural test. The environment-dependent flexural properties (including E-modulus and strength) are shown in Figure 8 and Table 4. The R-square value for each curve is also present in Figure 8, which indicates how well the curve fits test data points.

To be simplified, the predictive equation for the E-modulus degradation as the function of moisture content was curve fitted by the linear interpolant function, while for the flexural strength the exponential function was used. All the curve fitting processes were conducted by MATLAB R2011b, employing the least square method. The obtained predictive equation is as follows: 

*E*-modulus, 20 °C, absorption process:

*E* = −2725 × *M*_t_/*M*_∞_ + 16,609 (6)

*E*-modulus, 20 °C, absorption-desorption process:

*E* = −2428 × *M*_t_/*M*_∞_ + 16,333 (7)

Flexural strength, 20 °C, absorption process:

(8) $$S=103^{-(\frac{M_{t}}{M_{\infty }}-1.1)}+257$$

Flexural strength, 20 °C, absorption-desorption process:

(9) $$S=15.5^{-(\frac{M_{t}}{M_{\infty }}-1.87)}+242$$

*E*-modulus, 40 °C, absorption process:

*E* = −2795 × *M*_t_/*M*_∞_ + 15,409 (10)

*E*-modulus, 40 °C, absorption-desorption process:

*E* = −628 × *M*_t_/*M*_∞_ + 13,095 (11)

Flexural strength, 40 °C, absorption process:

(12) $$S=123^{-(\frac{M_{t}}{M_{\infty }}-1.04)}+221$$

Flexural strength, 40 °C, absorption-desorption process:

(13) $$S=3.23^{-(\frac{M_{t}}{M_{\infty }}-4.25)}+166$$

All the predictive curves are illustrated in Figure 8 for comparison with the experimental results.

For the specimens tested at 20 °C (Figure 8a), the E-modulus of GFRP laminates decreases gradually as the moisture content increases from fully dry to fully saturated. The E-modulus of the moisture saturated specimen is 14,022 MPa (as shown in Table 4), which is 15.6% lower than that of the unconditioned dry specimen. For the specimens with the moisture contents 30% and 50% of the saturation level, the loss of E-modulus is 4.4% and 9.5% respectively. With regard to specimens in the moisture desorption process, the E-modulus does not decrease significantly as compared to the specimens at the same moisture uptake level. Accordingly, the slight loss of E-modulus is 1.7%, 5.1% and 4.2% at the moisture uptake levels 0%, 30% and 50% respectively. In terms of flexural strength (see Figure 8b), there is a general exacerbation of decrease between the fully dry specimens and 30% moisture content specimens, regardless of the moisture absorption/desorption process. More than 20% loss of flexural strength is evident. After this stage, as the moisture uptake content increases, the flexural strength of specimens slightly decreases, until reaching 265 MPa for the moisture fully saturated specimens. At the end, the total drop of flexural strength is 35.4% of the fully dry specimens. Similar to the E-modulus degradation, the difference of flexural strength between absorption process and desorption process is very limited.

With regard to the environment-dependent flexural properties of GFRP laminates at 40 °C, the E-modulus of GFRP laminates regularly decreases from 15,409 MPa (dry) to 12,780 MPa (moisture fully saturated), and then slightly increases to 13,095 MPa after being fully dried in the moisture desorption process. Different from that at 20 °C, there is a significant unrecoverable loss (15.0%) of the E-modulus for the dry specimens tested at 40 °C. Accordingly, the loss of 11.1% and 6.1% of the E-modulus is evident for the specimens with 30% and 50% moisture content respectively in the desorption process. As to the flexural strength of specimens tested at 40 °C, a rapid decrease is observed at a moisture content of about 30% of moisture saturation level, and then the flexural strength slightly decreases to 214 MPa of the moisture fully saturated specimens, which is the lowest value among the whole series of tests. It is 42.9% lower than that of the unconditioned dry specimens (375 MPa, as listed in Table 4) tested at 40 °C, and 47.9% lower than that of the unconditioned dry specimens (410 MPa) tested at 20 °C. The most severe loss of flexural strength indicates that the combination of moisture and temperature effects can significantly influence the mechanical properties of GFRP laminates. Comparing the unconditioned dry specimens with the dry specimens after the moisture desorption, a decrease of 16.4% of the flexural strength is observed for the specimens tested at 40 °C. However, for the specimens with moisture contents 30% and 50%, the loss of flexural strength is not obvious. Even a slight increase is observed for the specimens with 30% moisture content in the moisture desorption process.

It is clear that the hot and wet environment seriously degrades the flexural properties of GFRP laminates, and in turn influences the durability of FRP composite structures. For the moisture effects, as stated in the researches [21,29,30], the absorbed moisture can cause both reversible and irreversible changes to the FRP composites, such as water plasticization and the disruption of hydrogen bonds between the molecular chains in the polymer. It is because the matrix often has hydrophilic groups that attract water molecules, which can form hydrogen bonds with water molecules via the hydroxyl groups [21]. Plasticization is a physical mechanism, which degrade the stiffness and strength of the polymer. It can be reversible at the initial stages of aging when the absorbed water is removed and no chemical reaction occurs. Furthermore, prolonged environmental exposure often leads to irreversible changes that induce permanent property alterations within the matrix, the fibre surfaces and the fibre/matrix interface. For instance, the polymer relaxation process is related to the polymer chain rearranging or redistribution of free-volume elements to provide additional sites of suitable size and accessibility to accommodate more penetrant molecules. It is a long-term and complicated process, which is a combination of chemical and physical reaction. Moreover, the fibre/matrix debonding, matrix cracking due to moisture/thermal cycles and ultimately fiber breaking would also contribute to the mechanical degradation. The fibre/matrix interface and the interphase region around fibres, are susceptible to deterioration due to moisture absorption. With the fibre/matrix debonding [31], there is enhanced interfacial capillary action along the fibres that can offer more space for moisture penetration and further promote their degradation. With regard to the temperature effects, when the test temperature is approaching the *T*_g_ of FRP composite materials, the mechanical performance such as E-modulus, strength and fatigue resistance significantly decreases [32,33]. Sometimes, the mass loss of FRP composites occurs following high-temperature exposure [28,34]. The recommended working temperature for FRP composite structures should be at least 20 °C lower than the *T*_g_ of FRP composite materials. Furthermore, the researches [33,35] confirmed that a decrease of *T*_g_ was evident when the moisture uptake content increased in the FRP composite materials. Meanwhile, as already proven in previous research [28], the high temperature can speed up the moisture diffusion process. Thus, the interaction between moisture and temperature effects accelerates the environmental degradation process on the FRP composite material, which in turn explains why the combination of moisture and temperature seriously deteriorates the mechanical properties of FRP materials.

## 4. Coupled Hygro-Mechanical FE Analysis

Generally, the environmental degradation experiments (mainly concerning moisture and temperature effects) for FRP composite materials are limited to a fairly short time, normally no more than 5 years. However, the expected service life of infrastructures such as bridges exceeds 50 years. Thus, the short-term experimental investigations are not sufficient to estimate the long-term performance of FRP structures. To achieve this aim, some accelerated experimental methods were developed by the researches [10,36,37,38], in which the temperature or atmospheric pressure of the environmental aging conditions were raised beyond the normal service conditions to accelerate the moisture diffusion and degradation process. These accelerating experimental methods were confirmed useful and time-effective to investigate the durability of FRP composites and adhesive materials. But, researches indicated that the high aging temperatures approaching glass transition temperature of specimens would improve the mechanical performance by post-cure or deteriorate the materials by inducing thermal cracks, which do not occur in the real use of FRP composite structures. Another method for studying the environmental degradation of mechanical behaviour of FRP materials and structures is the predictive FE modeling [39]. The first step is modeling moisture transport through FRP structures in order to determine the moisture concentration distribution through the cross-sections as a function of time. The material parameters required for the transient diffusion FE analysis are the diffusion coefficient and the solubility coefficient, which can be obtained from the short term gravimetric experiments, as stated in previous research [28]. And then, based on the obtained moisture concentration distribution, the environment-dependent mechanical behaviour of FRP structures can be investigated using the coupled hygro-mechanical FE analysis. The input moisture-dependent material properties of FRP composites are obtained by the material tests (such as flexural test, tensile test and short-beam shear test).

**Figure 9:**
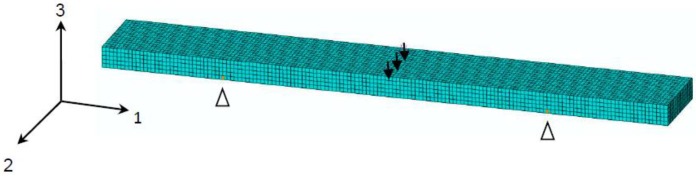
FE model of the flexural test specimen.

To develop the coupled hygro-mechanical FE modeling method, the FE model of the GFRP flexural test specimen was calibrated (as shown in Figure 9). The moisture diffusion process in the FRP specimen was modeled by the transient diffusion FE analysis (thoroughly described in previous research [28]) and validated by comparing with the gravimetric experimental results, as shown in Figure 5. From the moisture diffusion analysis, the moisture concentration distribution across the FRP specimen section was obtained as a function of time, which can be read into the stress analysis as a predefined field variable. The predictive equations (Equations (6), (7), (10) and (11)) of environment-dependent material properties served as the input of field-dependent material properties of FRP composites, which was obtained by the flexural tests. Hence, the E-modulus of each element was determined by the local moisture concentration. Thus, the coupled hygro-mechanical FE modeling was realized on the flexural test specimen. Subsequently, it can be employed to simulate other material tests (such as short-beam shear tests) and FRP structures. 

**Figure 10:**
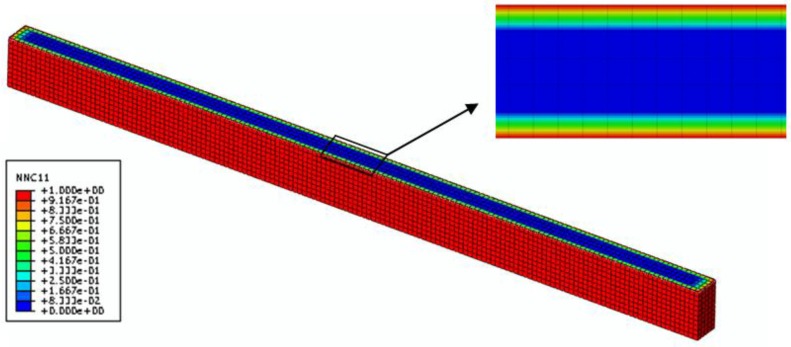
Nominal moisture concentration distribution across the mid-plane of the FRP specimenwith 30% moisture uptake content (time = 24 h).

**Figure 11:**
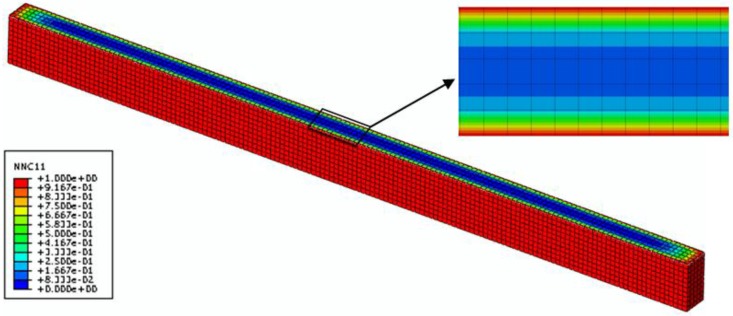
Nominal moisture concentration distribution across the mid-plane of the FRP specimen with 50% moisture uptake content (time = 229 h).

To validate this coupled hygro-mechanical FE modeling method, two groups of FRP specimens were selected: F-50%-20 °C and F-30%-40 °C-D. Firstly, the moisture concentration distributions throughout the mid-section of these two specimens are shown in Figure 10 and Figure 11. Based on these two predefined fields, the coupled hygro-mechanical FE analysis was executed. The FE stress analysis was linear-elastic. The element used was C3D8R, which is an 8-node linear brick element, with the capacity of reduced integration and hourglass control. In total 2000N load was applied at mid-span of the specimen.

**Figure 12:**
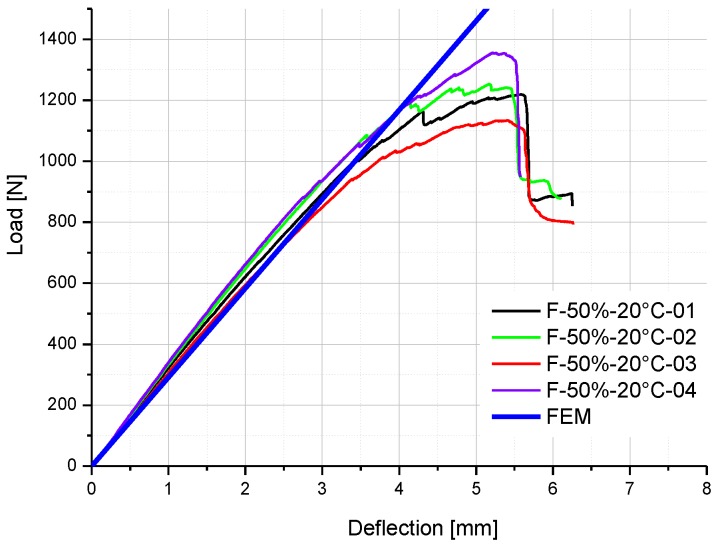
Comparison of experimental and FE results on the load-deflection curve of F-50%-20 °C specimens.

**Figure 13:**
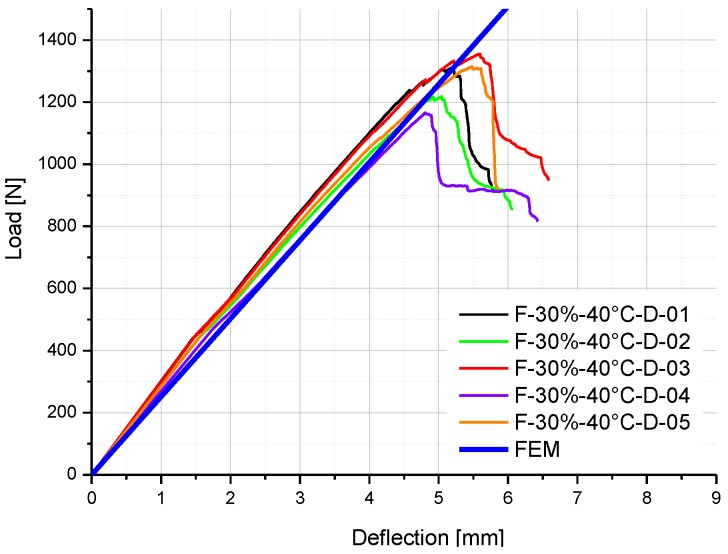
Comparison of experimental and FE results on the load-deflection curve of F-30%-40 °C-D specimens.

The comparisons of experimental results and FE results on the load-deflection curves of F-50%-20 °C specimens and F-30%-40 °C-D specimens are illustrated in Figure 12 and Figure 13 respectively. Good agreement is obtained in the linear stage, which means the environment-dependent stiffness of GFRP specimens with different moisture uptake contents can be predicted by the coupled hygro-mechanical FE model. Hence, the accuracy of the FE model is validated, and the methodology of the hygro-mechanical numerical analysis is proven.

## 5. Conclusions

This paper describes the investigation on the environment-dependent flexural properties of the FRP laminate material, which is achieved by the three-point bending tests according to the ASTM test code D790-10. The hydrothermal aging condition is a typical hot/wet aging environment (40 °C-water) for the application of FRP bridge decks. The test conditions vary in terms of test temperature, moisture uptake content and absorption/desorption process. The following conclusions can be drawn:

(1) For GFRP laminates tested at 20 °C, the *E*-modulus and flexural strength of moisture saturated GFRP laminates dropped 15.6% and 35.4% respectively, comparing with the fully dry materials. While, the difference of E-modulus and flexural strength between absorption process and desorption process was very limited.
(2) For GFRP laminates tested at 40 °C, the *E*-modulus and flexural strength of moisture saturated GFRP laminates dropped 17.1% and 42.9% respectively, comparing with the fully dry materials. There were significant unrecoverable losses of *E*-modulus (15.0%) and flexural strength (16.4%) for the GFRP laminates experienced both moisture absorption and desorption process.
(3) Experimental results confirmed that the combination of moisture and temperature seriously deteriorated the flexural properties of GFRP laminates, on both strength and stiffness.
(4) Predictive equations for environment-dependent flexural properties of FRP laminates were obtained by using the least square method for the curve fitting. These predictive equations can be employed as the input parameters for a coupled hygro-mechanical FE model, as well as a contribution to the design code as far as the long-term performance of FRP structures is concerned.
(5) A coupled hygro-mechanical FE modeling method was developed to analyze the environment-dependent mechanical behaviours of FRP laminates, and was validated by the experimental results of flexural tests. In the following research works, it could be employed in the parametric FE analysis, and predict the environment-dependent behaviours of other material tests or FRP structures.

## Figures and Tables

**Table 1:** Glass-fibre-reinforced polymer (GFRP) laminate properties of EQX1200 (supplied by Owenscorning, Toledo, OH, USA).

Product name	Total weight (g/m^2^)	Weight uniformity (g/m^2^)				
		Yarn roving				Knit yarn
		0° (Warp)	+45°	90° (Weft)	−45°	
EQX 1200	1,193	283	300	300	300	10

**Table 2:** Mechanical properties of GFRP laminates (supplied by Owenscorning, Toledo, OH, USA).

Property	Tensile (ISO 527-4)		Compression (ISO 8515)		Flexural (ISO 14.125)	
Mean value	Warp	Weft	Warp	Weft	Warp	Weft
Strength	331 MPa	314 MPa	220 MPa	200 MPa	473 MPa	433 MPa
Modulus	18 GPa	17 GPa	14 GPa	14 GPa	13 GPa	11 GPa

**Table 3:** GFRP laminate specimens for flexural tests.

Specimen identification		Test	*M*_t_/*M*_∞_	Test temperature	After desorption	Numder of specimens
Set-1	F-0%-20 °C	flexural	0	20 °C	no	5
	F-0%-40 °C	flexural	0	40 °C	no	5
Set-2	F-30%-20 °C	flexural	30%	20 °C	no	5
	F-30%-40 °C	flexural	30%	40 °C	no	5
Set-3	F-50%-20 °C	flexural	50%	20 °C	no	5
	F-50%-40 °C	flexural	50%	40 °C	no	5
Set-4	F-100%-20 °C	flexural	100%	20 °C	no	5
	F-100%-40 °C	flexural	100%	40 °C	no	5
Set-5	F-50%-20 °C-D	flexural	50%	20 °C	yes	5
	F-50%-40 °C-D	flexural	50%	40 °C	yes	5
Set-6	F-30%-20 °C-D	flexural	30%	20 °C	yes	5
	F-30%-40 °C-D	flexural	30%	40 °C	yes	5
Set-7	F-0%-20 °C-D	flexural	0	20 °C	yes	5
	F-0%-40 °C-D	flexural	0	40 °C	yes	5

**Table 4:** Flexural property degradation of GFRP laminates.

Specimen identification	E-Modulus * (MPa)	Standard deviation (MPa)	Flexural strength * (MPa)	Standard deviation (MPa)
F-0%-20 °C	16,609	386	411	6.89
F-0%-40 °C	15,409	852	375	8.49
F-30%-20 °C	15,873	867	299	8.88
F-30%-40 °C	13,874	500	249	11.42
F-50%-20 °C	15,038	710	269	17.03
F-50%-40 °C	13,870	535	252	17.57
F-100%-20 °C	14,022	514	265	13.74
F-100%-40 °C	12,780	538	214	6.08
F-50%-20 °C-D	14,408	551	260	15.46
F-50%-40 °C-D	13,019	832	239	17.85
F-30%-20 °C-D	15,059	740	324	37.04
F-30%-40 °C-D	12,336	166	277	9.85
F-0%-20 °C-D	16,333	204	410	10.80
F-0%-40 °C-D	13,095	698	314	13.92

* mean value of five specimens.
